# The International Standard Set of Outcome Measures for the Assessment of Hearing in People with Osteogenesis Imperfecta

**DOI:** 10.1097/MAO.0000000000003921

**Published:** 2023-06-16

**Authors:** Thadé Goderie, Sebastian Hendricks, Chiara Cocchi, I. Diane Maroger, Dagmar Mekking, Isabelle Mosnier, Angela Musacchio, David Vernick, Cas Smits

**Affiliations:** ∗ Department of Otolaryngology–Head and Neck Surgery, Ear and Hearing, Amsterdam UMC location Vrije Universiteit Amsterdam; † Amsterdam Public Health Research Institute, Amsterdam, the Netherlands; ‡ Department of Audiology and Audiovestibular Medicine, Sight and Sound Centre, Great Ormond Street Hospital for Children NHS FT, London, UK; § Department of Sensorial Organs, Audiology Operative Unit, Sapienza University of Rome, Rome Italy; ∥ Otolaryngology Department, University Hospital of Modena, Modena, Italy; ¶ French OI association patient representative, Paris, France; ∗∗ Care4BrittleBones Foundation, Wassenaar, the Netherlands; †† Technologies et thérapie génique pour la surdité, Institut de l'audition, Institut Pasteur/Inserm/Université Paris Cité, Paris, France—Unité Fonctionnelle Implants Auditifs, ORL, GH Pitié-Salpêtrière, AP-HP Sorbonne Université, Paris, France; ‡‡ Harvard Medical School, Beth Israel Lahey Hospital, Department of Surgery, Division of Otolaryngogy, Boston, Massachusetts; §§ Amsterdam UMC location University of Amsterdam, Otolaryngology–Head and Neck Surgery, Ear and Hearing, Amsterdam, the Netherlands.

**Keywords:** Clinician-reported outcome measures, Adult, Assessment, Audiology, Child, CROM, Follow-up, Patient-reported outcome measures, Pediatric, PROM

## Abstract

The aim is to recommend a minimum standard set of clinician-reported outcome measures (CROMs) and patient-reported outcome measures (PROMs) on hearing for people with osteogenesis imperfecta (OI). This project is part of the larger “Key4OI” project initiated by the “Care4BrittleBones foundation” of which the goal is to improve quality of life of people with OI. Key4OI provides a standard set of outcome measures and covers a large set of domains affecting the well-being of people with OI.

**Methods:**

An international team of experts in OI, comprising specialists in audiological science, medical specialists, and an expert patient representative, used a modified Delphi consensus process to select CROMs and PROMs to evaluate hearing problems in people with OI. In addition, focus groups of people with OI identified key consequences of their hearing loss. These criteria were matched to categories of preselected questionnaires to select a PROM that matched their specific hearing-related concerns best.

**Results:**

Consensus on PROMs for adults and CROMs for adults and children was reached. The focus of the CROMs was on specific audiological outcome measures and standardized follow-up.

**Conclusions:**

This project resulted in a clear consensus statement for standardization of hearing-related PROMs and CROMs and follow-up management of patients with OI. This standardization of outcome measurements will facilitate comparability of research and easier international cooperation in OI and hearing loss. Furthermore, it can improve standard of care in people with OI and hearing loss by incorporating the recommendations into care pathways.

## INTRODUCTION

Osteogenesis imperfecta (OI) is a rare genetic disorder also known as “brittle bone disease.” The estimated incidence is around 1:10.000 to 1:20.000 newborns, but incidence data vary worldwide (1). In 80 to 90% of cases, OI is associated with a heterozygous mutation in COL1A1 or COL1A2 genes, which encode for collagen type 1 and is essential for healthy bone formation (2,3). Other collagen-related genes with different inheritance patterns (i.e., autosomal dominant, autosomal recessive, X-linked recessive) are responsible for the remainder of OI cases (4). OI is classically divided into four types (type I to IV, “Sillence classification”) based on clinical presentation, radiographic features, and pattern of inheritance (5), and a fifth type was added in 2010 (6). Next-generation sequencing techniques are providing new insight into the disorder, and molecular genetic testing is now integrating the classical clinical description of OI patients (7). More recently, a genetic classification has been proposed (4). Clinical characteristics of OI include blue sclerae, short stature, bone fragility, muscle weakness, joint hypermobility, cardiopulmonary disorder, dentinogenesis imperfecta, and hearing loss (8). The clinical presentation and the progression of the disease show a high variability. A clear relation between phenotype and genetic mutations has not been proven so far (9).

Hearing loss can be present from childhood, but prevalence increases with age and differs per Sillence type of OI (10). The reported prevalence of hearing loss in the pediatric OI population has a wide range, from 0 to 77% (11,12). However, most pediatric case series show a lower prevalence of hearing loss compared with adults, and the hearing loss is partly explained by serous otitis media (11). The reported prevalence of hearing loss in adults has a wide range as well, but most larger cohorts report a prevalence of 28 to 58% with the most common age of onset between the second and fourth decade of life (11,13–15). Hearing loss can be conductive, sensorineural, or mixed (15). In a post-mortem study and surgical studies, fractures of middle ear ossicles, fixation of the stapes, and atrophic stapes crura were seen (16–19). Furthermore, the inner ear can show degenerative changes of the organ of Corti and spiral ganglion cells, and atrophy of the stria vascularis and hyalinization of the spiral ligament (16). In the Mov13 mouse, which serves as an animal model for Sillence type I OI, middle ear effusion with mucosal edema and infiltration of inflammatory cells and loss of hair cells were seen (20). Besides age and the OI type, no other risk factors for the development and progression of hearing loss have been identified. Larger datasets (possibly by international cooperation), longitudinal data analysis, and treatment analysis are needed to identify these factors. To facilitate this, a shared and rigorous standard set of outcome measures is required.

The clinical manifestations of OI vary widely and can affect many domains, such as mobility, self-care, and participation. The negative consequences of hearing loss can be larger for people with OI compared with people with an isolated hearing loss because it comes on top of other health-related problems. So, even when the impairment is identical, the participation restrictions (handicap) may be much larger. Given that OI is a rare disease, most studies include small patient populations with heterogeneity of outcome measures being used. This makes it hard to compare outcomes from different clinics and different countries. A standard set of outcome measures can allow for pooling of data to facilitate 1) decision-making between providers and patients, 2) quality improvement, 3) allow for learning together, including benchmarking across organizations, and 4) accelerate and focus research and clinical trials. In 2018, the Care4BrittleBones foundation initiated a project named Key4OI (www.key4OI.org) (21) to develop a standard set of clinician-reported outcome measures (CROMs) and patient-reported outcome measures (PROMs), covering a large set of domains affecting the well-being of people with OI using the methodology of the International Consortium of Health Outcome Measures (ICHOM) (22,23). At a later stage, hearing and pulmonary were included in the Key4OI standard outcome set. The Key4OI standard set has been accredited by ICHOM (21,23).

The present article reports the results of the Key4OI hearing initiative. The objective is to propose a standard set of hearing-related outcome measures, including follow-up measurements using the input from people with OI and an international group of healthcare professionals (audiologists and otologists) with expertise in OI.

## METHODS

A *three-step* modified Delphi technique was used to develop consensus on a minimal standard outcome set of CROMs and PROMs (24,25). The Delphi technique is an iterative multistage process that actively transforms opinion into group consensus (25). It is recommended for use in a healthcare setting as a reliable means of determining consensus for a defined clinical problem (25). This consensus must be based on data derived from all stakeholders involved in the care of individuals with OI and hearing loss, including the people with OI themselves. To achieve this, an international expert team was assembled comprising seven healthcare professionals, four in audiology and three in otology, and a patient representative of a European OI patient organization. Criteria to be part of the expert group were scientific and clinical experience with OI and being part of an OI expertise center. First, the director of the nongovernmental organization Care4BrittleBones (D.M.) reached out to experts who had published about OI and hearing in the last 10 years. In addition, recognized experts in this field (e.g., professionals in OI expertise centers) and patient organizations were asked for their interest. Second, we invited the potential participants to a kick-off call, in which the objectives and the project were explained. Thereafter, they needed to commit or step back. Video team meetings were held before and between each of the measurement rounds. The consensus process took place between March and July 2021. Consensus statements were rated anonymously by each team member on a 9-point Likert scale (i.e., 1 indicating “completely disagree” and 9 indicating “completely agree”). A rate of 1 to 3 in ≥80% of responses was considered “low agreement” and led to rejection of the consensus statement. A rate of 7 to 9 in ≥80% of responses was considered “high agreement” and led to acceptance of the consensus statement. If a consensus statement was inconclusive (i.e., neither rejected nor accepted), the consensus statement was rediscussed in a subsequent video meeting and rated again in the next Delphi round. In a subsequent meeting, the consensus statements were rediscussed. In some consensus statements, we reworded the consensus statement to improve clarity and rated the consensus statement again in a next Delphi round. The Delphi surveys were set up on a server that is used for scientific surveys. The access was restricted to the Care4BrittleBones organization as a project facilitator (not participant in the surveys). The data was collected and shared anonymously. The director of Care4BrittleBones (D.M.) coordinated the project, arranged and moderated video team meetings, and organized focus groups. The patient representative (I.D.M.) recruited other OI patients with hearing loss to participate in focus groups by contacting the European and American OI patient organizations. The expert team was not involved in recruiting the focus group and was not involved in focus group sessions because of ethical considerations. Overall, representatives of six countries on two continents took part in the expert team. One expert involved was a patient expert. Twenty-two OI patients from nine countries took part in the focus groups.

### Focus Groups, Systematic Review of the Literature, and Data Extraction

Three focus groups of participants with OI and hearing loss met in video meetings on March 2, 5, and 8, 2021. The approach to the focus group was virtual and global to generate input that is valid for OI patients in different cultures and healthcare systems. The chosen approach and tools ensured accessibility for people with hearing loss. The number of times a specific hearing-related theme was mentioned was indicated. Participants also flagged their personal top priorities regarding hearing-related burden with an exclamation mark. The 10 themes that were flagged most and came up most in discussions were chosen as key themes and were taken into consideration when choosing a PROM.

Key hearing domains were discussed and selected in a first meeting of the expert team. All CROMs related to hearing were listed at first, based on clinical expertise and a systematic review of the literature on hearing outcomes in patients with OI. Relevant PROMs were identified. To our knowledge, no hearing-related PROMs were used in OI research to date. Systematic reviews of Viergever et al. (26) and Powell et al. (27) were used to select PROMs. A systematic search on PubMed was performed to identify pediatric PROMs. In addition, “records identified through other sources” were added. This could be any other source, for example, journals not included in PubMed. This also included questionnaires suggested by the expert team. PROMs specifically designed for hearing aid or cochlear implant users were excluded as our goals were to select PROMs that could be used for the whole OI population who have hearing difficulties, regardless of if they use a hearing aid or cochlear implant. PROMs selected for evaluation needed to be translated in at least six languages to be considered as a key outcome measure. Full-text versions of all publications meeting the eligibility criteria were shared with the consensus panel.

## RESULTS

In the preparation sessions, key themes related to hearing loss that affect people with OI were identified based on literature and clinical experience. Key themes were standardization of audiological assessments and standardized follow-up on audiological assessments. Tinnitus, hyperacusis, and vestibular dysfunction were not included. Consensus statements were categorized in age groups. It was agreed to differentiate between the following age groups: adults, pediatric ≥4 years, and pediatric <4 years. In addition, an overall statement on the importance of genetic testing was added.

During the focus group sessions, 28 themes were considered. Of these 28 themes, 14 themes were discussed in depth within the focus groups and clustered into 10 key themes (Table 1). These 10 themes were subsequently summarized by two people who had attended all three sessions. As a final step, the summary of each topic under discussion was shared with all participants of the focus groups, and explicit approval was given that the summary was an accurate reflection of the discussion they took part in. Details on the input of the focus groups are included as a supplemental digital content (SDC) (SDC Text 1, http://links.lww.com/MAO/B664). In the preparation sessions of the expert team, key themes were compared with items in the preselected PROMs and discussed.

**TABLE 1:** Inputs collected from focus groups

Difficulties and Problems Attributed to Hearing Loss	Number of Participants that Marked the Topic as a Personal Problem	Number of Participants that Marked the Topic as a Personal Priority
Loss of conversation ease (hearing and speaking) (N)	18	5
Difficulty in accessing good medical care		
a) Difficulty or delay of getting surgery (N)	a) 9	a) 3
b) Not enough ear doctors/hearing specialists who are experts in OI (N)	b) 7	b) 1
Psychosocial impact		
a) Loss of social participation (N)	a) 9	a) 2
b) Progressive isolation (N)	b) 5	b) 4
c) Loss of self-confidence (N)	c) 3	c) 2
d) Sadness, depression (N)	d) 4	d) 0
Impact to education or career (N)	14	9
Loss or extra sensitivities to sounds/noise (N)	5	0
Loss of independence/challenge of adaptation (N)	4	2
Dizziness/loss of balance (N)	3	2
Tinnitus (N)	3	2
Problems related to hearing aids (N)	4	1
Mental tiredness—fatigue (N)	3	1

Twenty-two people with hearing loss and OI participated.

Results of the consensus statements are reported in Table 2. The response rate was 100% for all three Delphi rounds. The full text of the original consensus statements is provided as an SDC (SDC Text 2, http://links.lww.com/MAO/B665). The entire process resulted in the following recommendations for the assessment of hearing in people with OI.

**TABLE 2:** Results for each consensus statement in voting rounds 1, 2, and 3

Consensus Statement	Voting Round 1	Voting Round 2	Voting Round 3
PROMs, adult, recommended			
Hearing handicap inventory adults (Newman et al., 1990)	≥80% agreement (88%)	≥80% agreement (100%)*^a^*	NI
Abbreviated profile of hearing aid benefit (Cox and Alexander, 1995)	<80% agreement (38%)	NI	NI
Hearing handicap inventory for the elderly (Ventry and Weinstein, 1982)	<80% agreement (50%)	NI	NI
Hearing handicap inventory for the elderly—screening/shortened version (Lichtenstein et al., 1988)	<80% agreement (25%)	NI	NI
Amsterdam inventory for auditory disability and handicap (Kramer et al., 1998)	<80% agreement (38%)	NI	NI
Speech, spatial and qualities of hearing scale (Gatehouse and Noble, 2004)	<80% agreement (75%)	NI	NI
Spatial hearing questionnaire (Tyler et al., 2009)	<80% agreement (25%)	NI	NI
PROMs, pediatric, not recommended*^b^*			
Any specific PROMS for use with children	NI	NI	>80% agreement (100%)
Overall, recommended			
Genetic testing for everyone with OI	NI	≥80% agreement (100%)	The consensus statement was reworded after Delphi consensus panel discussion, >80% agreement (100%) after rewording
CROMs, adults, recommended			
Tympanometry	NI	≥80% agreement (100%)	NI
Acoustic reflexes	NI	≥80% agreement (100%)	NI
Pure tone audiometry (bone conduction and air conduction), up to 8 kHz	NI	≥80% agreement (100%)	NI
Speech recognition testing in quiet (and aided in people with hearing loss)	NI	≥80% agreement (88%)	The consensus statement was reworded after Delphi consensus panel discussion, >80% agreement (100%) after rewording
Only for adults with hearing loss speech in noise testing (aided), unaided is optional for this group, record normal/abnormal	NI	≥80% agreement (88%)	NI
CROMs, adults, not recommended*^b^*			
Speech in noise testing in adults with normal hearing	NI	≥80% agreement (88%)	NI
Wideband absorbance and wideband reflectance tympanometry	NI	≥80% agreement (100%)	NI
A specific speech in noise test	NI	≥80% agreement (88%)	NI
CROMs, pediatric ≥4 years, recommended	NI		NI
Tympanometry	NI	≥80% agreement (100%)	NI
Acoustic reflexes	NI	≥80% agreement (100%)	NI
Pure tone audiometry (bone conduction and air conduction), up to 8 kHz	NI	≥80% agreement (100%)	NI
Speech recognition in quiet (and aided in children with hearing loss)	NI	≥80% agreement (88%)	The consensus statement was reworded after Delphi consensus panel discussion, ≥80% agreement (100%) after rewording
Only for children with hearing loss, aided speech in noise testing, unaided is optional for this group, record normal/abnormal	NI	<80% agreement (75%)	≥80% agreement (88%)
CROMs, pediatric ≥4 years, not recommended*^b^*			
Speech in noise testing in children with normal hearing	NI	≥80% agreement (100%)	NI
Wideband absorbance and wideband reflectance tympanometry	NI	≥80% agreement (100%)	NI
A specific speech in noise	NI	≥80% agreement (88%)	NI
CROMs, pediatric <4 years			
Conditioned play audiometry or visual reinforcement audiometry dependent on cognitive abilities	NI	≥80% agreement (100%)	NI
Tympanometry	NI	≥80% agreement (100%)	NI
Acoustic reflexes	NI	≥80% agreement (100%)	NI
If ear-independent audiometry cannot be performed: otoacoustic emissions (only without diagnosed hearing loss)	NI	≥80% agreement (100%)	NI
If behavioral audiometry cannot be performed: ABR testing (air conduction and if elevated bone conduction)	NI	≥80% agreement (100%)	NI
Follow-up adults			
Adult without a hearing loss or with nonprogressive hearing loss: follow-up with a time interval of 5 years	NI	<80% agreement (50%)	≥80% agreement (88%)
Adult with progressive hearing loss or hearing aids: assess annually	NI	≥80% agreement (100%)	NI
Follow-up children	NI		
Regular newborn screening	NI	≥80% agreement (100%)	NI
No hearing loss: next assessment around the age of 3.5 and 5.5 years, thereafter every 3 years	NI	≥80% agreement (100%)	NI
If speech and language development is delayed, earlier assessment required	NI	≥80% agreement (100%)	NI
If progressive hearing loss or hearing aids: assess annually	NI	≥80% agreement (100%)	NI
If family, including first grade, is affected by profound hearing loss or a genetic mutation correlated with higher risk of hearing loss: assess annually	NI	≥80% agreement (100%)	NI

CROMS, clinician-reported ouctome measures; PROMS, patient-reported outcome measures.

Original consensus statements, including those that were reworded, can be read in SDC Text 2 (http://links.lww.com/MAO/B664). NI indicates not included in the voting round.

*^a^*In the second Delphi round, consensus was reached to use the HHIA instead of the RHHI.

*^b^*Agreement in the columns “CROMS or PROMS, not recommended” indicates that the consensus recommends to not include a specific CROM or PROM.

### PROMs

Seven PROMs were selected for evaluation (28-34). The “hearing handicap inventory for adults” (HHIA, 25 questions) (28) was the most recommended PROM. It is commonly used and fits well with the feedback from both the focus group and the experts. In the second Delphi round, the “revised hearing handicap inventory” (RHHI) (subset of 18 questions of the 25 questions) was discussed as a possible alternative to the HHIA (35). This version can be administered to adults of all ages and has improved discriminant validity on the subscales “emotional consequences” and “social/situational effects” and is more efficient because of the reduced number of questions. As the HHIA is currently used more frequently, we chose the HHIA as the standard PROM, but overtime, this may be changed to the RHHI if this becomes common practice.

We do not recommend any specific PROM for use with children with OI. The reasons are as follows: 1) there was no input from focus groups specific to children, 2) none of the experts in the group had experience using PROMs for children with OI related to hearing, and 3) no publications exist on hearing-related PROMs used in children with OI yet. However, considering that hearing loss deeply affects communicative abilities and language development in children, outcomes need to be established in an open dialogue with the family with the support of a multidisciplinary team (audiologists and speech language pathologist trained for children with hearing loss).

A key theme not included in the selected PROM but mentioned by 16 of 22 participants of the focus groups was “difficulty in accessing good medical care.” They reported difficulties in finding otolaryngologists and audiologists with both knowledge and experience in treating people with OI, which can lead to delays in getting optimal treatment.

### Clinician-reported outcome measures

In all age groups (i.e., adults, children ≥4 yr, and children <4 yr), tympanometry and acoustic reflex testing are recommended. Wideband absorbance and wideband reflectance are promising techniques but currently not recommended for the minimal standard set, as they are not widely available. In adults and children ≥4 years, pure tone audiometry is recommended, including air and bone conduction measurements up to 8 kHz. As a minimum standard, we recommend reporting bone and air conduction thresholds averaged across 0.5, 1, 2, and 4 kHz. In children <4 years, conditioned play audiometry or visual reinforcement audiometry is recommended, depending on cognitive abilities. If behavioral audiometry cannot be performed, otoacoustic emission measurements and auditory brainstem response (ABR) testing are recommended to estimate hearing thresholds. It is important to perform ABR at a young age to increase the chance of successful testing in natural sleep.

Speech recognition in quiet is recommended for adults and children ≥4 years. For hearing aid users, additionally aided speech recognition in quiet should be tested at a 50-dB hearing level or a speech level equivalent to approximately 65-dB sound pressure level. Aided speech-in-noise testing is recommended for adults and children ≥4 years; unaided testing is optional. As a large variety of speech-in-noise tests is available, we do not recommend a specific speech-in-noise test.

### Follow-up

Rules and schedules regarding neonatal hearing screening might be different depending on the country. If children pass the neonatal hearing screening, follow-up hearing assessment around the age of 3.5 and 5.5 years is recommended. It is recommended to perform the hearing assessment before starting preschool or primary school. Starting at the age of 3.5 years, ear-specific pure-tone audiometry can be performed reliably. If testers are unable to assess the child with pure-tone (play) audiometry, visual reinforcement audiometry should be performed. In case of a suspected hearing loss or delayed speech and language development, assessment at an age under 3.5 years is required. For children with a confirmed hearing loss, at least annual assessments in a pediatric audiological center are recommended. The audiological assessment can be set more frequently considering the progression of hearing loss in first-degree relatives.

Adults without a hearing loss or with nonprogressive hearing loss are recommended to have an audiological assessment every 5 years. In cases with a progressive hearing loss, testing should be done at least annually.

### Genetic Testing

The expert team recommends genetic testing for everyone with OI, as genetic factors may influence the occurrence or progression of hearing loss. This will also provide more data required for such research. The outcome of the genetic testing may support better hearing loss management through early recognition of “at risk” individuals.

### Follow-up after Middle Ear Surgery

Audiological assessment is recommended before and within 12 months after surgery.

## DISCUSSION

In this study, a Delphi consensus panel sought to provide a standardized set of PROMs and CROMs on hearing for people with OI. The current project on hearing is part of a larger project named Key4OI, which uses ICHOM methodology to create a standardized set of outcome measures on all domains of health, psychological, and daily functioning in people affected by OI (23). The goal was not to develop a clinical guideline but to recommend a standard set of outcome measures to enable comparison of care and improve quality and pathways of hearing care. By using the same outcome measures, the data from different centers can be combined, increasing statistical power. When the recommendations of the project on audiological testing and follow-up are broadly used, data from smaller centers can still be helpful by adding them to ongoing projects to achieve a larger sample size. This can be critical when studying rare conditions, such as OI.

As a sensory disability, hearing loss is often referred to as an “invisible disability.” Especially in people with OI who often have multiple health issues, hearing loss can be easily overlooked. In the focus group sessions, participants mentioned low awareness of the high prevalence of hearing loss in the OI community and among caregivers involved in supporting patients with OI. The reduced mobility of some people with OI can make speech understanding more challenging, as head orientation can affect speech intelligibility in noise (36). Focus group participants mentioned their concern about the difficulty of expressing themselves in emergency situations because of their hearing loss. This is even more relevant to the OI population with a higher incidence of hearing loss compared with the general population as they have a higher hospital admittance rate because of their OI (37). Focus groups also mentioned increased fatigue attributed to their hearing loss. In general, fatigue is increased in the OI population (38). Hearing loss can contribute to this fatigue as it poses an increased overall acoustic challenge requiring an increased cognitive demand, which is a key contributor to listening effort (39,40). By including hearing in the Key4OI program, hearing becomes part of a standardized set of health checks, and the project wishes it will no longer be overlooked.

Currently, a pilot is running for the original standard outcome set of Key4OI (hearing not included) in six different clinical care teams from different countries. The feasibility of implementing Key4OI in a clinical setting as well as a research setting is analyzed. One of the features of the Key4OI project is a standardized “checklist” with topics relevant to people with OI, such as pain, fatigue, anxiety, and much more. All this information flows into a treatment plan for the coming year(s). Hearing is part of this checklist, and follow-up and treatment are part of this plan. By measuring hearing in all patients with OI, clinics involved in research on OI will develop a much better idea of the prevalence of hearing loss in OI and subtypes of OI. We aim to create an increased awareness of minimal hearing healthcare among people involved in the care for people with OI. Furthermore, this might be a start to develop an international guideline for hearing healthcare for people with OI.

### Strengths and Limitations

The strength of this study is that the consensus statements were developed based on input by a variety of hearing health care professionals, including audiologists and otologists. In addition, by using the input from focus groups, a PROM could be selected based on items that are important for people in the OI community with a hearing loss.

The limitation of the study is the geographic spread of the expert team, which only involves professionals from Europe and North America. Specific expert experience in other regions was thus not considered in the development of the consensus statements. The number of participants in the expert team was eight. This relatively low number of participants was due to limited research available on hearing and OI, which limited the number of people we could approach with apparent clinical and research experience in hearing and OI. However, “the Delphi group size does not depend on statistical power, but rather on group dynamics for arriving at consensus among experts” (41). No parents of children with OI and hearing loss took part in the focus groups. Outcome measures on vestibular function were not considered. Vestibular dysfunction is rarely studied in patients with OI, but a study by Kuurila et al. (42) reported vertigo in 52% of patients with OI in a population of 42 patients. The authors would like to see further research on vestibular dysfunction especially as this can increase the chance of falling with subsequent fractures due to the high bone fragility. Tinnitus (reported by 3 of 22 participants of the focus groups) and hyperacusis were considered separate problems, albeit related to hearing loss, and were not part of the current project. Because no data are available that tinnitus and hyperacusis present differently in people with OI, standard counseling and treatments are recommended.

## CONCLUSION

The scope of this study was to develop consensus statements for standardization of PROMs and CROMs and follow-up management of people with hearing loss and OI. The consensus statements establish a standardized approach and should improve the quality of care for both children and adults with OI. It can also facilitate research and international cooperation in OI and hearing loss by allowing pooling of data from multiple centers to give a more accurate picture of outcomes. Further research is required to identify risk factors associated with hearing loss, to predict the progression of hearing loss, and to optimize treatment for people with OI.

## Supplementary Material
