# Supplementary material for: The International Standard Set of Outcome Measures for the Assessment of Hearing in People with Osteogenesis Imperfecta
Source: Otol Neurotol. 2023 Jun 16;44(7):e449–55. doi: 10.1097/MAO.0000000000003921 (PMC10348656; doi:10.1097/MAO.0000000000003921)
Supplement: Supplementary file 1 [file on-44-e449-s001.docx]

**ABSTRACT**

**Objective:**

The aim is to recommend a minimum standard set of clinical reported outcome measures (CROMs) and patient reported outcome measures (PROMs) on hearing for people with Osteogenesis Imperfecta (OI). This project is part of the larger “Key4OI” project initiated by the “Care4BrittleBones foundation” of which the goal is to improve quality of life of people with OI. Key4OI provides a standard set of outcome measures and covers a large set of domains affecting the well-being of people with OI.

**Methods:**

An international team of experts in OI, comprising specialists in audiological science, medical specialists, and an expert patient representative, used a modified Delphi consensus process to select CROMs and PROMs to evaluate hearing problems in people with OI. In addition, focus groups of people with OI identified key consequences of their hearing loss. These criteria were matched to categories of pre-selected questionnaires to select a PROM which matched their specific hearing related concerns best.

**Results:**

Consensus on PROMs for adults and CROMs for adults and children were reached. The focus of the CROMs was on specific audiological outcome measures and standardized follow-up.

**Conclusions:**

This project resulted in a clear consensus statement for standardization of hearing related PROMs and CROMs and follow-up management of patients with OI. This standardization of outcome measurements will facilitate comparability of research and easier international cooperation in OI and hearing loss. Further, it can improve standard of care in people with OI and hearing loss by incorporating the recommendations into care pathways.

**Introduction**

Osteogenesis imperfecta (OI) is a rare genetic disorder also known as “brittle bone disease”. The estimated incidence is around 1:10.000 to 1:20.000 newborns, but incidence data vary worldwide (1). In 80-90% of cases, OI is associated with a heterozygous mutation in COL1A1 or COL1A2 genes which encode for collagen type 1 and is essential for healthy bone formation (2,3). Other collagen-related genes with different inheritance patterns (i.e., autosomal dominant, autosomal recessive, X-linked recessive) are responsible for the remainder of OI cases (4). OI is classically divided into 4 types (type I to IV, “Sillence classification”) based on clinical presentation, radiographic features and pattern of inheritance (5) and a fifth type was added in 2010 (6). Next-generation sequencing techniques are providing new insight into the the disorder, and molecular genetic testing is now integrating the classical clinical description of OI patients (7). More recently, a genetic classification has been proposed (4). Clinical characteristics of OI include blue sclerae, short stature, bone fragility, muscle weakness, joint hypermobility, cardiopulmonary disorder, dentinogenesis imperfecta, and hearing loss (8). The clinical presentation and the progression of the disease show a high variability. A clear relation between phenotype and genetic mutations has not been proven so far (9).

Hearing loss can be present from childhood but prevalence increases with age and differs per Sillence type of OI (10). The reported prevalence of hearing loss in the pediatric OI population has a wide range, from 0 to 77% (11,12). However, most pediatric case series show a lower prevalence of hearing loss compared to adults and the hearing loss is partly explained by serous otitis media (11). The reported prevalence of hearing loss in adults has a wide range as well, but most larger cohorts report a prevalence of 28-58% with the most common age of onset between the second and fourth decade of life (11,13-15). Hearing loss can be conductive, sensorineural or mixed (15). In a post-mortem study and surgical studies fractures of middle ear ossicles, fixation of the stapes, and atrophic stapes crura were seen (16-19). Further, the inner ear can show degenerative changes of the organ of Corti and spiral ganglion cells, and atrophy of the stria vascularis and hyalinization of the spiral ligament (16). In the Mov13 mouse, which serves as an animal model for Sillence type I OI, middle ear effusion with mucosal edema and infiltration of inflammatory cells and loss of hair cell was seen (20). Besides age and OI type, no other risk factors for the development and progression of hearing loss have been identified. Larger datasets (possibly by international cooperation), longitudinal data analysis, and treatment analysis are needed to identify these factors. To facilitate this, a shared and rigorous standard set of outcome measures is required.

The clinical manifestations of OI vary widely and can affect many domains, such as mobility, self-care and participation. The negative consequences of hearing loss can be larger for people with OI compared to people with an isolated hearing loss because it comes on top of other health related problems. So, even when the impairment is identical, the participation restrictions (handicap) may be much larger. Given that OI is a rare disease, most studies include small patient populations with a heterogeneity of outcome measures being used. This makes it hard to compare outcomes from different clinics and different countries. A standard set of outcome measures can allow for pooling of data to 1) improve decision making between providers and patients, 2) facilitate quality improvement, 3) allow for learning together, including benchmarking across organizations, and 4) accelerate and focus research and clinical trials. In 2018 the Care4BrittleBones foundation initiated a project, named Key4OI (www.key4OI.org) (21), to develop a standard set of clinical reported outcome measures (CROMs) and patient reported outcome measures (PROMs), covering a large set of domains affecting the well-being of people with OI using methodology of the International Consortium of Health Outcome Measures (ICHOM) (22,23). At a later stage, hearing and pulmonary were included in the Key4OI standard outcome set. The Key4OI standard set has been accredited by ICHOM (21,23).

The present paper reports the results of the Key4OI hearing initiative. The objective is to propose a standard set of hearing related outcome measures, including follow-up measurements using the input from people with OI and an international group of healthcare professionals (audiologists and otologists) with expertise in OI.

**Methods**

A *three-step* modified Delphi technique was used to develop consensus on a minimal standard outcome set of clinical reported outcome measures (CROMs) and patient reported outcome measures (PROMs) (24,25). The Delphi technique is an iterative multi-stage process to actively transform opinion into group consensus (25). It is recommended for use in a healthcare setting as a reliable means of determining consensus for a defined clinical problem (25). This consensus must be based on data derived from all stakeholders involved in the care of individuals with OI and hearing loss, including the people with OI themselves. To achieve this, an international expert team was assembled comprising 7 healthcare professionals, 4 in audiology and 3 in otology, and a patient representative of a European OI patient organization. Criteria to be part of the expert group were scientific and clinical experience with OI and being part of an OI expertise center. First, the Director of the non-governmental organization Care4BrittleBones (DM) reached out to experts who had published about OI and hearing in the last 10 years. In addition, recognised experts in this field (e.g., professionals in OI expertise centers) and patient organisations were asked for their interest. Second, we invited the potential participants to a kick off call, in which the objectives and the project were explained. Thereafter, they needed to commit or step back. Video team meetings were held prior to and between each of the measurement rounds. The consensus process took place between March and July 2021. Consensus statements were rated anonymously by each team member on a nine-point Likert scale (i.e. 1 indicating “completely disagree” and 9 indicating “completely agree”). A rate of 1-3 in ≥ 80% of responses was considered “low agreement” and led to rejection of the consensus statement. A rate of 7-9 in ≥ 80% percent of responses was considered “high agreement” and led to acceptance of the consensus statement. If a consensus statement was inconclusive (i.e., not rejected nor accepted) the consensus statement was re-discussed in a subsequent video meeting and rated again in the next Delphi round. In a subsequent meeting, the consensus statements were re-discussed. In some consensus statements, we reworded the consensus statement to improve clarity and rated the consensus statement again in a next Delphi round. The Delphi surveys were set up on a server which is used for scientific surveys. The access was restricted to the Care4BrittleBones organization as a project facilitator (not participant in the surveys). The data was collected and shared anonymously. The director of Care4BrittleBones (DM) coordinated the project, arranged and moderated video team meetings and organized focus groups. The patient representative (IDM) recruited other OI patients with hearing loss to participate in focus groups by contacting the European and American OI patient organizations. The expert team was not involved in recruiting the focus group and was not involved in focus group sessions, because of ethical considerations. Overall, representatives of six countries on two continents took part in the expert team. One expert involved was a patient expert. Twenty-two OI patients from 9 countries took part in the focus groups.

**Focus groups, systematic review of the literature, and data extraction.**Three focus groups of participants with OI and hearing loss met in video meetings on March 2^nd^, 5^th^, and 8^th^, 2021. The approach to the focus group was virtual and global, to generate input that is valid for OI patients in different cultures and healthcare systems. The chosen approach and tools ensured accessibility for people with hearing loss. The number of times a specific hearing-related theme was mentioned was indicated. Participants also flagged their personal top priorities regarding hearing-related burden with an exclamation mark. The ten themes that were flagged most and came up most in discussions were chosen as key themes and were taken into consideration when choosing a PROM.

Key hearing domains were discussed and selected in a first meeting of the expert team. All CROMs related to hearing were listed at first, based on clinical expertise and a systematic review of the literature on hearing outcomes in patients with OI. Relevant PROMs were identified. To our knowledge, no hearing related PROMs were used in OI research to date. Systematic reviews of Viergever, et al. (26) and Powell, et al. (27) were used to select PROMs. A systematic search on PubMed was performed to identify pediatric PROMs. In addition, “records identified through other sources” were added. This could be any other source, e.g., journals not included in PubMed. This also included questionnaires suggested by the expert team. PROMs specifically designed for hearing aid or cochlear implant users were excluded as our goals were to select PROMs which could be used for the whole OI population who have hearing difficulties, regardless of if they use a hearing aid or cochlear implant. PROMs selected for evaluation needed to be translated in at least 6 languages to be considered as a key outcome measure. Full-text versions of all publications meeting the eligibility criteria were shared with the consensus panel.

**Results**In the preparation sessions, key themes related to hearing loss that affect people with OI were identified based on literature and clinical experience. Key themes were standardization of audiological assessments and standardized follow-up on audiological assessments. Tinnitus, hyperacusis, and vestibular dysfunction were not included. Consensus statements were categorized in age groups. It was agreed to differentiate between the following age groups: adults, pediatric ≥4 years, and pediatric <4 years. In addition, an overall statement on the importance of genetic testing was added.

During the focus group sessions, 28 themes were considered. Of these 28 themes, 14 themes were discussed in depth within the focus groups and clustered into 10 key themes (Table 1). These 10 themes were subsequently summarized by 2 people who had attended all 3 sessions. As a final step, the summary of each topic under discussion was shared with all participants of the focus groups and explicit approval was given that the summary was an accurate reflection of the discussion they took part in. Details on the input of the focus groups are included as supplemental digital content (SDC), (SDC Text 1, http://links.lww.com/MAO/B664). In the preparation sessions of the expert team, key themes were compared to items in the pre-selected PROMs and discussed.

Results of the consensus statements are reported in Table 2. The response rate was 100% for all three Delphi rounds. Full-text of the original consensus statements is provided as SDC (SDC Text 2, http://links.lww.com/MAO/B665). The entire process resulted in the following recommendations for the assessment of hearing in people with OI.

*PROMs*

The “hearing handicap inventory for adults” (HHIA, 25 questions) (28) is the most recommended PROM. It is commonly used and fits well with the feedback from both the focus group and experts. In the second Delphi round the “revised hearing handicap inventory” (RHHI) (subset of 18 questions of the 25 questions) was discussed as a possible alternative to the HHIA (29). This version can be administered to adults of all ages and has improved discriminant validity on the subscales “emotional consequences” and “social/situational effects” and is more efficient due to the reduced number of questions. As the HHIA is currently used more frequently we chose the HHIA as standard PROM but overtime this may be changed to the RHHI if this becomes common practice.
We do not recommend any specific PROM for use with children with OI. Reasons are: (a) there was no input from focus groups specific to children, (b) none of the experts in the group had experience using PROMs for children with OI related to hearing, and (c) no publications exist on hearing related PROMs used in children with OI yet. However, considering that hearing loss deeply affects communicative abilities and language development in children, outcomes need to be established in an open dialogue with the family with the support of a multidisciplinary team (audiologists and speech language pathologist trained for children with hearing loss).
A key theme not included in the selected PROM but mentioned by 16 out of 22 participants of the focus groups was “difficulty in accessing good medical care”. They reported difficulties in finding otolaryngologists and audiologists with both knowledge and experience in treating people with OI, which can lead to delays in getting optimal treatment.

*CROMs*In all age groups (i.e., adults, children ≥ 4 years, and children < 4 years), tympanometry and acoustic reflex testing are recommended. Wideband absorbance and wideband reflectance are promising techniques but currently not recommended for the minimal standard set, as they are not widely available. In adults and children ≥ 4 years, pure tone audiometry is recommended, including air and bone conduction measurements up to 8 kHz. As a minimum standard, we recommend reporting bone and air conduction thresholds averaged across 0.5, 1, 2, and 4 kHz. In children < 4 years, conditioned play audiometry or visual reinforcement audiometry is recommended, depending on cognitive abilities. If behavioral audiometry cannot be performed, otoacoustic emission (OAE) measurements and auditory brainstem response (ABR) testing is recommended to estimate hearing thresholds. It is important to perform ABR at a young age to increase the chance of successful testing in natural sleep.

Speech recognition in quiet is recommended for adults and children ≥ 4 years. For hearing aid users, additionally aided speech recognition in quiet should be tested at 50 dB hearing level or a speech level equivalent to approximately 65 dB SPL. Aided speech-in-noise testing is recommended for adults and children ≥ 4 years; unaided testing is optional. As a large variety of speech-in-noise tests is available, we do not recommend a specific speech-in-noise test.

*Follow-up*

Rules and schedules regarding neonatal hearing screening might be different depending on the country. If children pass the neonatal hearing screening, follow-up hearing assessment around the age of 3.5 and 5.5 years is recommended. It is recommended to perform the hearing assessment before starting preschool or primary school. Starting at the age of 3.5 years, ear-specific pure-tone audiometry can be performed reliably. If testers are unable to assess the child with pure-tone (play) audiometry, visual reinforcement audiometry should be performed. In case of a suspected hearing loss or delayed speech and language development, assessment at an age under 3.5 years is required. For children with a confirmed hearing loss, at least annual assessments in a pediatric audiological center are recommended. The audiological assessment can be set more frequently considering the progression of hearing loss in first-degree relatives.

Adults without a hearing loss or with non-progressive hearing-loss are recommended to have an audiological assessment every 5 years. In cases with a progressive hearing loss, testing should be done at least annually.

*Genetic testing*

The expert team recommends genetic testing for everyone with OI, as genetic factors may influence the occurrence or progression of hearing loss. This will also provide more data required for such research. The outcome of the genetic testing may support better hearing loss management through early recognition of “at risk” individuals.

*Follow-up after middle ear surgery*

Audiological assessment is recommended before and within 12 months after surgery.

**Discussion**

In this study, a Delphi consensus panel sought to provide a standardized set of PROMs and CROMs on hearing for people with OI. The current project on hearing is part of a larger project named Key4OI which uses ICHOM methodology to create a standardized set of outcome measures on all domains of health, psychological, and daily functioning in people affected by OI (23). The goal was not to develop a clinical guideline but to recommend a standard set of outcome measures to enable comparison of care and improve quality and pathways of hearing care. By using the same outcome measures, the data from different centers can be combined, increasing statistical power. When the recommendations of the project on audiological testing and follow-up are broadly used, data from smaller centers can still be helpful by adding it to ongoing projects to achieve a larger sample size. This can be critical when studying rare conditions, such as OI.

As a sensory disability, hearing loss is often referred to as an “invisible disability.” Especially in people with OI who often have multiple health issues, hearing loss can be easily overlooked. In the focus group sessions, participants mentioned a low awareness of the high prevalence of hearing loss in the OI community and amongst caregivers involved in supporting patients with OI. The reduced mobility of some people with OI can make speech understanding more challenging, as head orientation can affect speech intelligibility in noise (30). Focus group participants mentioned their concern about the difficulty of expressing themselves in emergency situations because of their hearing loss. This is even more relevant to the OI population with a higher incidence of hearing loss compared to the general population as they have a higher hospital admittance rate because of their OI (31). Focus groups also mentioned increased fatigue attributed to their hearing loss. In general, fatigue is increased in the OI population (32). Hearing loss can contribute to this fatigue as it poses an increased overall acoustic challenge requiring an increased cognitive demand, which is a key contributor to listening effort (33,34). By including hearing in the Key4OI program, hearing becomes part of a standardized set of health checks and the project wishes it will no longer be overlooked.

Currently, a pilot is running of the original standard outcome set of Key4OI (hearing not included) in six different clinical care teams from different countries. The feasibility of implementing Key4OI in a clinical setting as well as a research setting is analyzed. One of the features of the Key4OI project is a standardized “checklist” with topics relevant to people with OI, such as pain, fatigue, anxiety, and much more. All this information flows into a treatment plan for the coming year(s). Hearing is parts of this checklist and follow-up and treatment are part of this plan. By measuring hearing in all patients with OI, clinics involved in research on OI will develop a much better idea of the prevalence of hearing loss in OI and subtypes of OI. We aim to create an increased awareness of minimal hearing healthcare among people involved in the care for people with OI. Furthermore, this might be a start to develop an international guideline for hearing healthcare for people with OI.

*Strengths and limitations*

A strength of this study is that the consensus statements were developed based on input by a variety of hearing health care professionals, including audiologists and otologists. In addition, by using the input from focus groups, a PROM could be selected based on items that are important for people in the OI community with a hearing loss.

A limitation of the study is the geographic spread of the expert team, which involves professionals from only Europe and North America. Specific expert experience in other regions was thus not considered in the development of the consensus statements. The number of participants in the expert team was 8. This relatively low number of participants was due to limited research available on hearing and OI, which limited the number of people we could approach with apparent clinical and research experience in hearing and OI. But, “the Delphi group size does not depend on statistical power, but rather on group dynamics for arriving at consensus among experts” (35). No parents of children with OI and hearing loss took part in the focus groups. Outcome measures on vestibular function were not considered. Vestibular dysfunction is rarely studied in patients with OI, but a study of Kuurila, et al. (36) reported vertigo in 52% of patients with OI in a population of 42 patients. The authors would like to see further research on vestibular dysfunction especially as this can increase the chance of falling with subsequent fractures due to the high bone fragility. Tinnitus (reported by 3/22 participants of the focus groups) and hyperacusis were considered separate problems, albeit related to hearing loss, and were not part of the current project. Because no data is available that tinnitus and hyperacusis present differently in people with OI, standard counseling and treatments are recommended.

**Conclusion**

The scope of this study was to develop consensus statements for standardization of PROMs and CROMs and follow-up management of people with hearing loss and OI. The consensus statements establish a standardized approach and should improve the quality of care for both children and adults with OI. It can also facilitate research and international cooperation in OI and hearing loss by allowing pooling of data from multiple centers to give a more accurate picture of outcomes. Further research is required to identify risk factors associated with hearing loss, to predict the progression of hearing loss, and to optimize treatment for people with OI.

References

1. Martin E, Shapiro JR. Osteogenesis imperfecta:epidemiology and pathophysiology. *Curr Osteoporos Rep* 2007;5:91-7.

2. Bardai G, Moffatt P, Glorieux FH, et al. DNA sequence analysis in 598 individuals with a clinical diagnosis of osteogenesis imperfecta: diagnostic yield and mutation spectrum. *Osteoporos International* 2016;27:3607-13.

3. Forlino A, Marini JC. Osteogenesis imperfecta. *Lancet* 2016;387:1657-71.

4. Marini JC, Forlino A, Bächinger HP, et al. Osteogenesis imperfecta. *Nat Rev Dis Primers* 2017;3:17052.

5. Sillence DO, Senn A, Danks DM. Genetic heterogeneity in osteogenesis imperfecta. *Journal of medical genetics* 1979;16:101-16.

6. Warman ML, Cormier-Daire V, Hall C, et al. Nosology and classification of genetic skeletal disorders: 2010 revision. *Am J Med Genet A* 2011;155a:943-68.

7. Van Dijk FS, Sillence DO. Osteogenesis imperfecta: clinical diagnosis, nomenclature and severity assessment. *Am J Med Genet A* 2014;164a:1470-81.

8. Marom R, Rabenhorst BM, Morello R. Osteogenesis imperfecta: an update on clinical features and therapies. *European journal of endocrinology* 2020;183:R95-R106.

9. Marini JC, Dang Do AN. Osteogenesis Imperfecta. *Endotext*. South Dartmouth (MA): MDText.com, Inc. Copyright © 2000-2022, MDText.com, Inc., 2000.

10. Machol K, Hadley TD, Schmidt J, et al. Hearing loss in individuals with osteogenesis imperfecta in North America: Results from a multicenter study. *Am J Med Genet A* 2020;182:697-704.

11. Carré F, Achard S, Rouillon I, et al. Hearing impairment and osteogenesis imperfecta: Literature review. *Eur Ann Otorhinolaryngol Head Neck Dis* 2019;136:379-83.

12. Joseph JK, Maharaj SH. Osteogenesis Imperfecta and hearing loss in the paediatric population. *Int J Pediatr Otorhinolaryngol* 2021;150:110914.

13. Paterson CR, Monk EA, McAllion SJ. How common is hearing impairment in osteogenesis imperfecta? *J Laryngol Otol* 2001;115:280-2.

14. Kuurila K, Kaitila I, Johansson R, et al. Hearing loss in Finnish adults with osteogenesis imperfecta: a nationwide survey. *Ann Otol Rhinol Laryngol* 2002;111:939-46.

15. Swinnen FKR, Dhooge IJM, Coucke PJ, et al. Audiologic Phenotype of Osteogenesis Imperfecta: Use in Clinical Differentiation. *Otology & Neurotology* 2012;33.

16. Santos F, McCall AA, Chien W, et al. Otopathology in Osteogenesis Imperfecta. *Otol Neurotol* 2012;33:1562-6.

17. Skarzynski H, Kordowska K, Skarzynski PH, et al. Results of stapedotomy in otosurgical treatment of adult patients with osteogenesis imperfecta. *Auris Nasus Larynx* 2019.

18. Swinnen FK, De Leenheer EM, Coucke PJ, et al. Audiometric, surgical, and genetic findings in 15 ears of patients with osteogenesis imperfecta. *Laryngoscope* 2009;119:1171-9.

19. Hijazi M, Mihailescu SD, Horion J, et al. Stapes surgery in osteogenesis imperfecta: retrospective analysis of 18 operated ears. *Eur Arch Otorhinolaryngol* 2021;278:4697-705.

20. Stankovic KM, Kristiansen AG, Bizaki A, et al. Studies of otic capsule morphology and gene expression in the Mov13 mouse--an animal model of type I osteogenesis imperfecta. *Audiol Neurootol* 2007;12:334-43.

21. Key4OI. Available at: https://www.key4oi.org/.

22. Nijhuis W, Franken A, Ayers K, et al. A standard set of outcome measures for the comprehensive assessment of osteogenesis imperfecta. *Orphanet J Rare Dis* 2021;16:140.

23. International Consortium for Health Outcomes Measurement I. Available at: https://www.ichom.org/standard-sets/#methodology.

24. Dalkey N, Helmer O. An Experimental Application of the Delphi Method to the Use of Experts. *Management Science* 1963;9:458-67.

25. Boulkedid R, Abdoul H, Loustau M, et al. Using and reporting the Delphi method for selecting healthcare quality indicators: a systematic review. *PLoS One* 2011;6:e20476.

26. Viergever K, Kraak JT, Bruinewoud EM, et al. Questionnaires in otology: a systematic mapping review. *Syst Rev* 2021;10:119.

27. Powell J, Powell S, Robson A. A systematic review of patient-reported outcome measures in paediatric otolaryngology. *J Laryngol Otol* 2018;132:2-7.

28. Newman CW, Weinstein BE, Jacobson GP, et al. The Hearing Handicap Inventory for Adults: psychometric adequacy and audiometric correlates. *Ear Hear* 1990;11:430-3.

29. Cassarly C, Matthews LJ, Simpson AN, et al. The Revised Hearing Handicap Inventory and Screening Tool Based on Psychometric Reevaluation of the Hearing Handicap Inventories for the Elderly and Adults. *Ear Hear* 2020;41:95-105.

30. Grange JA, Culling JF. The benefit of head orientation to speech intelligibility in noise. *J Acoust Soc Am* 2016;139:703-12.

31. Kolovos S, Javaid MK, Pinedo-Villanueva R. Hospital admissions of patients with osteogenesis imperfecta in the English NHS. *Osteoporosis international : a journal established as result of cooperation between the European Foundation for Osteoporosis and the National Osteoporosis Foundation of the USA* 2021;32:1207-16.

32. Harsevoort AGJ, Gooijer K, van Dijk FS, et al. Fatigue in adults with Osteogenesis Imperfecta. *BMC Musculoskelet Disord* 2020;21:6.

33. Zekveld AA, Kramer SE, Festen JM. Cognitive load during speech perception in noise: the influence of age, hearing loss, and cognition on the pupil response. *Ear Hear* 2011;32:498-510.

34. Peelle JE. Listening Effort: How the Cognitive Consequences of Acoustic Challenge Are Reflected in Brain and Behavior. *Ear Hear* 2018;39:204-14.

35. Okoli C, Pawlowski SD. The Delphi method as a research tool: an example, design considerations and applications. *Information & Management* 2004;42:15-29.

36. Kuurila K, Kentala E, Karjalainen S, et al. Vestibular dysfunction in adult patients with osteogenesis imperfecta. *Am J Med Genet A* 2003;120A:350-8.
