# Supplementary material for: The International Standard Set of Outcome Measures for the Assessment of Hearing in People with Osteogenesis Imperfecta
Source: Otol Neurotol. 2023 Jun 16;44(7):e449–55. doi: 10.1097/MAO.0000000000003921 (PMC10348656; doi:10.1097/MAO.0000000000003921)
Supplement: Supplementary file 2 [file on-44-e449-s002.docx]

**Key Themes identified across sessions:**

1. **Loss of conversation ease (Hearing and Speaking)** Mentions: N=18
   Flagged as personal top priority: N=5
2. **Difficulty to access good medical care**
   - **Difficulty or delay of getting surgery** Mentions: N=9
     Flagged as personal top priority: N=3
   - **Not enough ear doctors and other hearing specialists knowing about OI** Mentions: N=7
     Flagged as a personal top priority: N=1
3. **Psychosocial impact**
   - **Loss of social participation** Mentions: N=9
     Flagged as personal top priority: N=2
   - **Progressive isolation** Mentions: N=5
     Flagged as personal top priority: N=4
   - **Loss of self-confidence** Mentions: N=3
     Flagged as personal top priority: N=2
   - **Sadness, Depression** Mentions: N=4
     Flagged as personal top priority: N=0
4. **Impact to education or career** Mentions: N=14
   Flagged as personal top priority: N=9
5. **Loss or extra sensitivities to sounds / noise** Mentions: N=5
   Flagged as personal top priority: N=0
6. **Loss of independence / Challenge of adaptation** Mentions: N=4
   Flagged as personal top priority: N=2
7. **Dizziness / Loss of balance** Mentions: N=3
   Flagged as personal top priority: N=2
8. **Tinnitus** Mentions: N=3
   Flagged as personal top priority: N=2
9. **Problems related to hearing aids** Mentions: N=4
   Flagged as a personal top priority: N=1
10. **Mental tiredness – Fatigue** Mentions: N=3
    Flagged as personal top priority: N=1

**CHALLENGES — PRIORITY TOPICS IDENTIFIED (in order of urgency)**

The following topics are descriptions of challenges experienced and described by the OI focus groups. These descriptions have been changed as little as possible to reflect the opinions and concerns of the OI focus groups and not of the authors of this article.

1. Loss of conversation ease (Hearing and speaking)

- It is important to be close to hear well;
- Need to see lips (worn masks are a problem);
- Difficulty to turn the neck to see who is speaking for some people with OI.
- When speech rate is too fast, especially in social situations like meals with many people.
- Fast changing speakers is a problem.
- Difficulty to understand in noise and understanding foreign languages and foreign accents.
  Learning languages becomes difficult and travelling alone no longer possible.
- 3 reports being told they “speak too loud”.

2. Difficulty to get proper surgery or treatment + Lack of qualified doctors and other hearing specialists about OI

- Surgery is complicated by our bone condition. Doctors don’t pick it up, don’t know what’s possible. Some got unnecessary surgery which brought no help. Others didn’t get surgical options and were given hearing aids. Cochlear Implant, when you have OI, is less likely to work (cochlea and temporal bone porous, electrical current can pass through and stimulate the facial nerve). Otospongiosis/osteroarthritis can cause occasional failure of stapes surgery.
- In general: There is a lack of doctors and specialists qualified in hearing and OI. Lack of testing at all ages. “*My audiologist doesn’t believe my OI has caused hearing loss and it’s been hard to get the right fit hearing aid due to size/shape of my ear canal”*
  Two OI patients report medical bias : “*I have never seen a Dr that knows about hearing loss in OI. The ones that I have seen do not understand additional implications that go with it - e.g. not being able to distinguish between noises. Also I have found it hard to get correct fitting inserts that don't cause my ears to become really watery*” ; *“why do you need your hearing sorted when you don’t do this or that (presumes I have no social life, don’t work etc)?”* Others mention lack of knowledge on accessibility on the part of doctors and medical staff dealing with OI.

3. Psychosocial impact

- Loss of social participation, leading to progressive isolation: most participants report suffering from psychosocial impact & shame of not being able to participate in cultural or social events. “*I lock up inside myself when there is too much noise*”. “*Trying to give an answer but answering wrong*’, so not contributing in friendly gatherings, staying silent.
- Loss of self-confidence: issue with partner and/or family, such as having to sleep with the hearing aid on, in order to care for baby. Problem of self-acceptance due to wearing hearing aid; children or teens with O.I can be teased. With age people learn to manage that impact better.
- Depression: Not asking for help anymore. Progressive nature:  Conversation difficulties -> loss of participation -> less likely to reach out, even to a psychologist -> isolation. To “get help” is tiring, as it is also linked to communicating.

4. Impact to education or career

- Can lead to leaving a job or position because of not being to participate in team meetings, boards, roundtables. Need to ask for help by another attendee, and therefore lose autonomy on the job. Others needed to change their choice of studies, give up studying, or change jobs, therefore not being able to use their talent. There is a challenge with passing job interviews. Problem to concentrate.
- It can be hard to hear colleagues who speak in low voice or whisper (eg. teaching job). Participants reported fear of losing one’s job due to not picking up information. Cost of live captions. Difficulties working in groups and having to follow video or information that is told collectively: barrier to cultural participation for OI person in the cultural field. Some people with OI and increasing hearing loss were forced to retire early.
- Difficulty using a telephone or audio device: is mentioned across all groups for its impact at work. Causes misunderstandings. “*The worse is with strangers who do not know of the hearing issues*”; “*Working as a receptionist in a noisy environment”,* or having to transcribe audio, forced OI people to change jobs as there was impact on the quality of their work.

5. Loss of independence / Challenge of adaptation

With age it may be more difficult to acquire more skills (lip reading or signing). You need to stay informed on new options to help with hearing. To learn and be informed on technology.

Lack of (knowledge about) accessibility is deplored by OI people who are not connected to the deaf community. “*Our main challenge is mobility, so hearing loss gets less attention in the OI and medical community*”.

6. Loss or extra sensitivities to sound / noise

- Extra-sensitivity to loud sounds or music is reported by 3 out of 22 participants.
- With hearing loss sounds become weaker and difficult to recognize or locate. 2 signal danger when crossing the road with wheelchair or crutches, not hearing cars properly.

7. Dizziness

For some this relates to tinnitus. But mostly due to vestibular vertigo. OI causes an issue when it comes to treating vertigo with an osteopath or manipulation of the neck because our necks are fragile. Vertigo can appear after CI surgery.

(Not directly related to hearing: when vertigo or dizziness results from basilar invagination; loss of balance in relation to hyper laxity of joints.)

8. Tinnitus

Causes sleep problems, headaches, bad mood. It makes you nervous. “When tinnitus is bad, I get dizzy and disoriented.

9. Problems related to hearing aids

- Difficulty finding hearing aid fitting our ear-ducts: “*Can’t find earplugs that fit for hearing aid*”.
- 2 report danger of having to remove hearing aid in medical care situations: OI person is more vulnerable to misunderstanding with the hospital staff— eg. in case of fracture.
- Ear infections reduced the benefit of hearing aids. BAHA helped.
- Having to remove hearing aid to partake in wellbeing activities in humid environment (spa, water games) makes you lose chances to enjoy it.

10. Tiredness / Mental fatigue: Many feel tired of concentrating all the time, by tinnitus, or stress.

**Initial list of all challenges related to hearing loss (in random order):**

- Need for surgery
- Dizziness
- Extra Sensitivity to noise
- Learning to speak
- Ear infections
- Speaking to loud
- Loss of conversation ease
- Tinnitus
- Perceiving and recognizing sounds
- Using a telephone (audio)
- Difficulty to get treatment (eg. stapedectomy, CI)
- Progressive Isolation
- Cost of treatment (insurance covering 1 hearing aid)
- Social acceptance as a child (teasing)
- Lack of accessibility
- Risk related to Air Travel
- Loss of social participation
- Feeling sad
- Loss of self-confidence
- Depression
- Challenge of adaptation
- Impact in a medical situation
- Difficulty to understand people with masks
- Feeling anxious
- Mental fatigue
- Lack of knowledge about OI and hearing in general
- Increase of dependence
- Not enough ear doctors and other hearing specialists knowing about OI]

**‘HOPE’ TOPICS IDENTIFIED (order of priority):**

1. More ear/hearing specialists knowing about OI. Not just in specific cities.

Better medical expertise, transfer of knowledge on OI and hearing. *“This should improve social and employment issues”*

2. More resources

- About tools, technology and treatments/ access to the technology that goes with the hearing aids; about ways to facilitate communication (cued speech, transcription tools, captions). “*The deaf community has access to better information*”
- About research in terms of advancement / progress in this field
- About OI and hearing loss

3. More peer support or mentoring about OI and hearing loss.

- There is not enough "OI & hearing" information for people with OI. The OI community talks more about fractures.
- We need access to the technology that goes with the hearing aid
- Need to share experiences with others who have OI on how to solve their problems and live a fulfilling life. Focus is on issues concerning children. Equal attention should be given to ‘adult issues’.

4. Psychological follow up with trained and accessible psychologist.

5. Societal hope:

- Scientific progress

Progress in the social representation of hearing loss (invisible disability). “*We need more social understanding about hearing loss. It’s a taboo that should be lifted (often source of comedy, hurtful*)”.

Some participants also expressed personal needs:

“*I need people to speak louder and slower. I wish I could concentrate better*”

“*My wishes would be to find a suitable device and perhaps another surgery*”

“*Having a hearing aid that is completely adaptable to what is needed (eg for social life etc.).  Personalized hearing aid*” ; “*Finding an auditory balance*”.
