# Supplementary material for: The International Standard Set of Outcome Measures for the Assessment of Hearing in People with Osteogenesis Imperfecta
Source: Otol Neurotol. 2023 Jun 16;44(7):e449–55. doi: 10.1097/MAO.0000000000003921 (PMC10348656; doi:10.1097/MAO.0000000000003921)
Supplement: Supplementary file 3 [file on-44-e449-s003.docx]

### Topics for the Care4BrittleBones Delphi Hearing Round 1, 2 and 3

### Delphi Hearing - Round 1

# Title: Key4OI Plus for Hearing in OI – Round 1

1. Abbreviated profile of hearing aid benefit

2. Hearing Handicap Inventory for the elderly

3. Hearing Handicap Inventory for the elderly - screening/shortened version

4. Hearing Handicap Inventory Adults (HHIA)
*(Reworded in Delphi round 2)*

5. Amsterdam Inventory for Auditory Disability and Handicap

6. Speech, Spatial and Qualities of hearing scale (SSQ)

7. Spatial hearing questionnaire

### Delphi Hearing Round 2

**Title: Key4OI Plus for Hearing in OI - Round 2**

**Overall**

1. The team recommends genetic testing for everyone with OI, as genetic factors seem to influence the occurrence or progression of hearing loss. The team acknowledged that more research in this field is required and this would be enabled through general genotype-phenotype matching.
*(Reworded in Delphi round 3)*

**PROMs Adults**

2. HHIA (25 questions) is recommended as PROM as it is commonly used and a very good fit with both the focus group feedback and feedback from practicing experts. Overtime this may be reduced to the “revised hearing handicap inventory” (subset of 18 questions of the 25 questions), if this becomes common practice.

**CROMs Adults**

**The following assessments are recommended by the Project Expert team for OI adults with and without hearing loss**

3. Tympanometry

4. Acoustic reflex thresholds

5. Speech recognition testing in quiet (aided in people with hearing loss). Speech recognition testing should assess speech understanding (%) at 60 decibel SPL
*(Reworded in Delphi round 3)*

6. Pure tone audiometry (bone conduction and air conduction), including 8 kHz (frequencies for bone conduction based on calibration)

7. In addition ONLY for OI adults with hearing loss, speech in noise testing (aided) is required. Unaided is optional for this group. For recording purposes simply noting normal / abnormal is sufficient as any local validated approach may be used, dependant of the language such as for example the Matrix Sentence Test or the HINT.

**The project team does currently NOT recommend the following as minimum standards for adults**

8. Speech in noise testing in adults with OI with normal hearing if no other issues are identified,

9. Wideband absorbance and wideband reflectance tympanometry due to the limited availability

10. A specific speech in noise test (HINT, or Matrix Sentence test) as the test would need to be available in all languages and usable for all clinics. For this reason, any locally available validated test is acceptable

**CROMS Children 4 years and older**

**The following assessments are recommended by the Project Expert team - for OI children 4 years and above with and without diagnosed hearing loss**

11. Pure tone (FM tone) audiometry (bone conduction and air conduction), including 8 kHz if possible

12. Speech recognition testing (in quiet / in noise) (aided in people with hearing loss). Speech recognition testing should assess speech understanding (%) at 60 decibel SPL

13. Tympanometry

14. Acoustic reflex thresholds

15. Only for children with hearing loss, aided speech in noise testing. Unaided is optional for this group. For recording purposes simply noting normal / abnormal is sufficient as any local validated approach may be used, dependant of the language such as for example the Matrix Sentence Test or the HINT.

16. Speech recognition testing in quiet (AND aided in children with hearing loss)

**The project team does currently NOT recommend to include into the recommended minimum standard for children**

17.

Speech in noise testing in children with normal hearing if no other issues are identified

18. Wideband absorbance and wideband reflectance tympanometry due to the limited availability

19. A specific speech in noise test (Hint, or Matrix Sentence test) as the test would need to be available in all languages and usable for all clinics. For this reason, any locally available validated test is acceptable

**CROMS Children younger than 4 years**

**The following assessments are recommended by the Project Expert team - for OI children younger than 4 years with and without diagnosed hearing loss**

20. Conditioned play audiometry or visual reinforcement audiometry dependent on cognitive abilities

21. Tympanometry

22. Acoustic reflex thresholds

23. If ear independent audiometry cannot be performed: Otoacoustic emission testing

24. If behavioural audiometry cannot be performed: electrophysiological assessment e.g., auditory brainstem response testing (ABR)- air conduction and if elevated bone conduction, or auditory steady state response audiometry (ASSR)

**Follow up Adults**

25. Adult without a hearing loss or with non-progressive hearing loss: follow-up with a time-interval of 5 years
*(Reworded in Delphi round 3)*

26. Adult with progressive hearing loss or hearing aids: minimum annual assessment

**Follow up Children**

27. Regular new-born screening

28. No hearing loss: Next assessment around the age of 3,5 years and 5,5 years, thereafter every 3 years

29. If speech and language development are delayed, earlier assessment required

30. If progressive hearing loss or hearing aids: minimum annual assessment

31. If family, including first grade, are affected by profound hearing loss or a genetic mutation correlated with higher risk of hearing loss, the child needs to be seen annually too ("red flag")

**Follow up Middle Ear Surgery**

32. Same as for hearing aid fitting: Use same standards before and after the surgery. The assessment after the surgery needs to be performed within 1 year after surgery

### Delphi Hearing Round 3

**Title: Key4OI Plus for hearing in OI - Round 3**

**Overall**

1. The team recommends genetic testing for everyone with OI, as genetic factors seem to influence the occurrence or progression of hearing loss. The team acknowledged that more research in this field is required and this would be enabled through general genotype-phenotype matching.

**PROMS Children**

2. We do not recommend any specific PROMS for use with children with OI. Reasons are:
(1) there was no input from focus groups specific to children,
(2) none of the experts in the group had personal experience using good validated PROMS for children with OI related to hearing and
(3) there are no publications on PROMS used in children with OI yet. The outcomes in children need to be established in a normal clinical dialogue with parents or child depending on age.

**CROMS Adults**

**The following assessments are recommended by the Project Expert team - for OI adults with and without hearing loss**

3. Unaided speech recognition testing in quiet and in people with hearing loss additionally aided speech recognition in quiet. Speech recognition testing should assess speech understanding (%) at 50 dBHL or a speech level equivalent to approximately 65 dBSPL

**CROMS Children 4 years and older**

**The following assessments are recommended by the Project Expert team - for OI children 4 years and above with and without diagnosed hearing loss**

4. Unaided speech recognition testing in quiet and in people with hearing loss additionally aided speech recognition in quiet. Speech recognition testing should assess speech understanding (%) at 50 dBHL or a speech level equivalent to approximately 65 dBSPL

5. Speech recognition testing in noise (aided in people with hearing loss)

**Follow up Adults**

6. Adults without a hearing loss or with non-progressive hearing loss: follow-up with a time-interval of 5 years unless there are hearing problems. In that case, the person needs to be seen earlier / as soon as possible.

OI: Osteogenesis Imperfecta

PROM: Patient Reported Outcome Measure

CROM: Clinical Reported Outcome Measure
